# Social media as a determinant of health

**DOI:** 10.1093/eurpub/ckae029

**Published:** 2024-02-24

**Authors:** Amrit Kaur Purba, Anna Pearce, Marion Henderson, Martin McKee, S Vittal Katikireddi

**Affiliations:** MRC/CSO Social and Public Health Sciences Unit, University of Glasgow, Glasgow, UK; MRC/CSO Social and Public Health Sciences Unit, University of Glasgow, Glasgow, UK; School of Social Work and Social Policy, University of Strathclyde, Glasgow, UK; London School of Hygiene and Tropical Medicine, London, UK; MRC/CSO Social and Public Health Sciences Unit, University of Glasgow, Glasgow, UK

Communication has transformed drastically, with couples glued to screens in restaurants and the emergence of ‘influencers’ and ‘fake news’ shaping daily life. By 2022, approximately 4.62 billion people, roughly 58% of the global population, used social media across various platforms.^1^ Despite widespread usage, a ‘digital divide’ in internet access persists, leaving many, even in affluent nations, ‘digitally excluded’ due to limited internet access. 

While social media offers health-related benefits, it also poses challenges, such as the promotion of unhealthy commodities and the spread of dis/misinformation, as seen throughout the coronavirus disease 2019 (COVID-19) pandemic.^2^ We argue that social media, a ubiquitous aspect of modern communication, is a major determinant of health, due to the profound impact it has on population health. As such, we urge health professionals and researchers to grasp its complexities.

We explore the potential mechanisms through which social media may impact health, demonstrated via a logic model presented in Supplementary figure S1.

## Improving the flow of health information

Social media has democratized health information dissemination, offering diverse content for both the public and professionals. Endorsed by the World Health Organisation and UNICEF, this adaptable approach allows information to be tailored to users' contexts, languages and cultural backgrounds. By being able to access and share health information, the public can easily engage with health professionals to co-develop management regimes. Social media’s benefits also extend to research and public monitoring and address traditional surveillance system limitations. However, concerns persist regarding representativeness, privacy, accuracy and personal data exposure to digital platforms or governmental surveillance agencies.

## Social connectedness and interpersonal relationships

Considerable evidence supports the positive impact of ‘social capital’ on health, particularly among minority groups.^3^ Intense relationships within close networks strengthen social connections crucial for individual well-being. The pandemic highlighted social media's role in connecting people with shared interests, transcending geographical barriers. However, social media networks can become ‘echo chambers’ for extreme and health-damaging messages. Moreover, the impact of social media on social capital depends on usage patterns; passive engagement lacks benefits seen in active engagement, while excessive use may increase loneliness compared to moderate use.

## Self-esteem and social comparison

Social media use is associated with mental health conditions such as anxiety and depression, attributable in part to its attention-economy business model and design features promoting addictive behaviours.^4^ Features like the ‘infinite scroll’ exploit the human desire for intermittent variable rewards, leading to self-esteem erosion, heightened social comparison and obsessive–compulsive behaviours. It is unlikely, tools like Instagram's ‘Take A Break’ and TikTok's opt-out screentime limits will effectively address this issue.

## Health behaviours

Extensive research documents the adverse effects of digital marketing, particularly influencer marketing, on the consumption of unhealthy commodities, contributing to global disease.^5^^,^^2^ Manufacturers pay influencers substantial sums to exploit their influence on purchasing decisions. Beyond this, users are influenced by the behaviours of friends in their social media network, impacting engagement in health-related behaviours.^2^ Concerns arise from exposure to such content and the evolving ranking algorithms shaping users' feeds based on previous behaviour, which are potentially promoting unhealthy content. Unhealthy commodity industries resist regulation, endorsing voluntary codes and lobbying against statutory measures, especially influencer marketing legislation. Despite general advertising regulations, influencers often neglect to disclose contractual relationships, leaving users unaware of potential conflicts of interest.

## Uptake of health services and guidance

Ranking algorithms contribute to the spread of dis/misinformation by prioritizing controversial issues that attract attention, often manipulated by non-human bots. These algorithms influence individual thinking, undermining trust in science and public institutions, and in some cases, leading to lower adherence to public health recommendations, as demonstrated by COVID-19’s ‘infodemic’.

Dis/misinformation can also amplify racism, sexism and xenophobia, with direct health consequences, impeding global efforts to advance healthcare and human rights. In the context of the climate emergency, dis/misinformation poses a concerning obstacle to necessary action with far-reaching health implications.

## Political and public health discourse

Social media's impact on civic engagement and democracy, as seen in recent elections, presents both positive and negative aspects. Online ‘echo chambers’ contribute to confirmation biases, reinforce extreme/polarized views and amplify misinformation. Platforms like Meta advocate self-regulation to counter misinformation but their response lacks transparency and effectiveness and ignores the organizations responsible for producing and promoting misleading information.

Disinformation strategies, involving bots and trolls, distort the online public health discourse, particularly in discussions on vaccine effectiveness. Identifying these entities without specialized knowledge is challenging, raising concerns about who is really behind these activities and the failure of social media corporations to accept any responsibility for undermining public health messaging.

## Recommendations

Social media should be considered a major determinant of health, providing various benefits but also posing risks, notably through the dissemination of dis/misinformation undermining public health messaging. Health professionals require new skills, and research into social media’s implications, heightened practitioner awareness of its opportunities and challenges and advocacy for strengthened regulation and legislation is crucial.

## Supplementary Material

ckae029_Supplementary_Data
