# Supplementary material for: Social media as a determinant of health
Source: Eur J Public Health. 2024 Feb 24;34(3):425–6. doi: 10.1093/eurpub/ckae029 (PMC11161142; doi:10.1093/eurpub/ckae029)
Supplement: ckae029_Supplementary_Data [file ckae029_supplementary_data.zip › ckae029_Supplementary_Data/ejph-2024-02-ed-0087-File002.docx]

# Supplementary Material: Social Media as a Determinant of Health

Dr Amrit Kaur Purba, A.Purba.1@research.gla.ac.uk

Dr Anna Pearce, Anna.Pearce@glasgow.ac.uk

Professor Marion Henderson, Marion.Henderson@strath.ac.uk

Professor Martin McKee, Martin.Mckee@lshtm.ac.uk

Professor S Vittal Katikireddi, Vittal.Katikireddi@glasgow.ac.uk

## Figure-1. Logic model illustrating pathways between social media and health

***
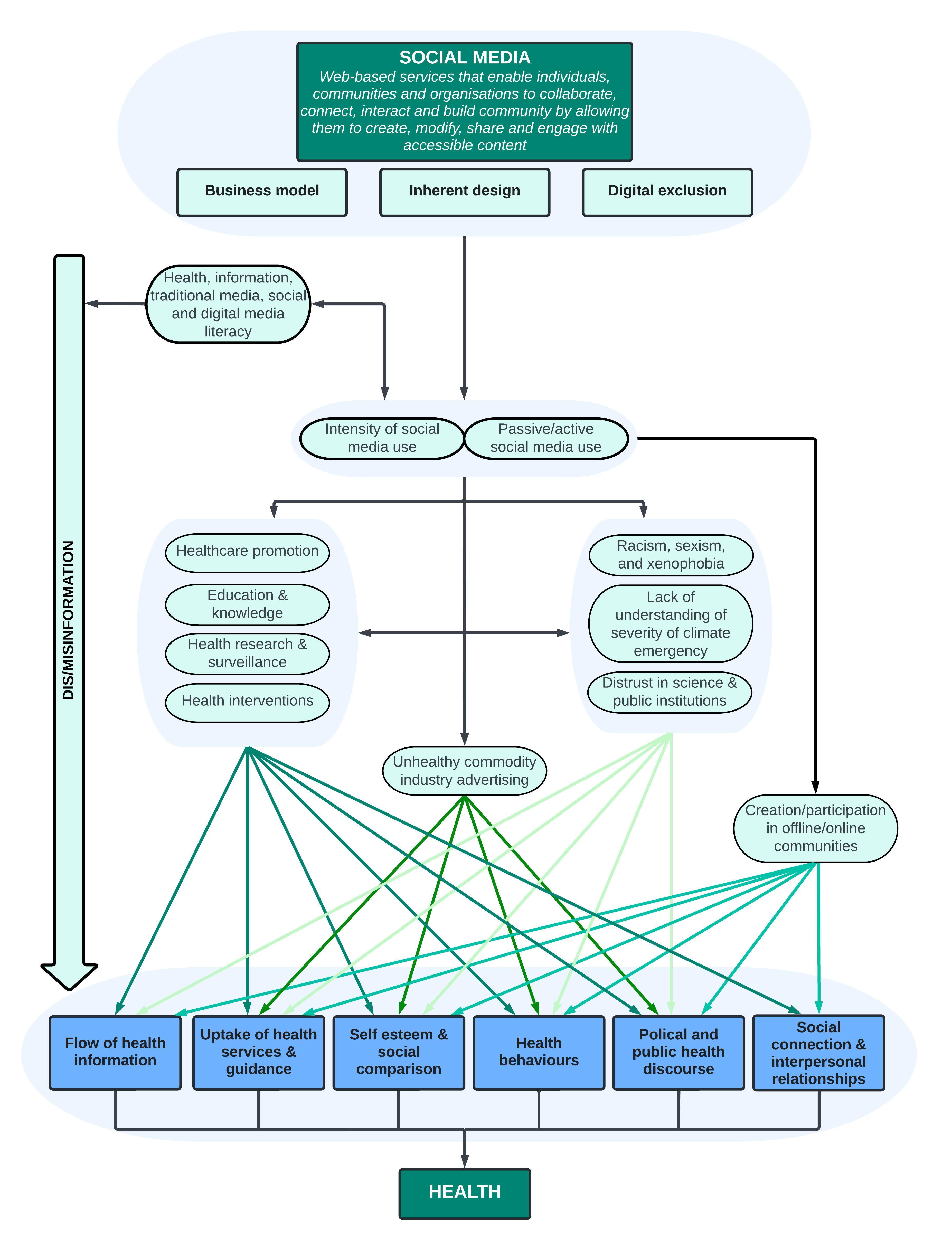
***

## Additional references

Mendoza-Herrera K, Valero-Morales I, Ocampo-Granados ME, et al. An overview of social media use in the field of public health nutrition: Benefits, scope, limitations, and a Latin American experience. *Prev Chronic Dis*. 2020;17(200047). DOI: 10.5888/PCD17.200047

Broniatowski DA, Jamison AM, Qi SH, AlKulaib L, et al. Weaponized health communication: Twitter bots and Russian trolls amplify the vaccine debate. *Am J Public Health*. 2018;108(10):1378–84. DOI: 10.2105/AJPH.2018.304567

Sinnenberg L, Buttenheim AM, Padrez K, et al. Twitter as a tool for health research: A systematic review. *Am J Public Health*. 2016;107(1):e1–8. DOI: 10.2105/AJPH.2016.303512

Aiello AE, Renson A, Zivich PN. Social media- and internet-based disease surveillance for public health. *Annu Rev Public Health*. 2019;41:101–18. DOI: 10.1146/ANNUREV-PUBLHEALTH-040119-094402

Winstone L, Mars B, Haworth CMA, et al. Social media use and social connectedness among adolescents in the United Kingdom: A qualitative exploration of displacement and stimulation. *BMC Public Health*. 2021;21(1). DOI: 10.1186/S12889-021-11802-9

Harris T. The slot machine in your pocket [Internet]. 2016 [accessed 04 February, 2023]. Available from: https://www.spiegel.de/international/zeitgeist/smartphone-addiction-is-part-of-the-design-a-1104237.html

Bhargava VR, Velasquez M. Ethics of the attention economy: the problem of social media addiction. *Bus Ethics Q*. 2020;31(3):1–39. DOI: 10.1017/BEQ.2020.32

5Rights Foundation. Too little too late? Instagram’s latest changes and what they will mean for kids [Internet]. 2022 [accessed 04 January, 2023]. Available from: https://5rightsfoundation.com/in-action/too-little-too-late-instagrams-latest-changes-and-what-they-will-mean-for-kids.html

Schultz W. Dopamine reward prediction error coding. *Dialogues Clin Neurosci*. 2016;18(1):23–32. DOI: 10.31887/DCNS.2016.18.1/WSCHULT

Gerritsen S, Sing F, Lin K, et al. The timing, nature and extent of social media marketing by unhealthy food and drinks brands during the COVID-19 pandemic in New Zealand. *Front Nutr*. 2021;8:645349. DOI: 10.3389/FNUT.2021.645349

Zenone M, Kenworthy N, Maani N. The social media industry as a commercial determinant of health. *Int J Heal Policy Manag*. 2022;(x):1–4. DOI: 10.34172/IJHPM.2022.6840

Lencucha R, Thow AM. Intersectoral policy on industries that produce unhealthy commodities: Governing in a new era of the global economy? *BMJ Glob Heal*. 2020;5(8).

Hinchliffe S. Online advertising programme – UK Government proposes increased regulatory oversight of online ads [Internet]. 2022 [accessed 04 January, 2023]. Available from: https://www.cliffordchance.com/briefings/2022/03/online-advertising-programme---uk-government-proposes-increased-.html

Carah N, Brodmerkel S. Alcohol marketing in the era of digital media platforms. *J Stud Alcohol Drugs*. 2021;82(1):18–27. DOI:10.15288/JSAD.2021.82.18

Think Tank European Parliament. The impact of influencers on advertising and consumer protection in the Single Market [Internet]. 2022 [accessed 04 August, 2022]. Available from: https://www.europarl.europa.eu/thinktank/en/document/IPOL_STU(2022)703350

Törnberg P. Echo chambers and viral misinformation: Modeling fake news as complex contagion. *PLoS One*. 2018;13(9):e0203958–e0203958. DOI: 10.1371/JOURNAL.PONE.0203958

World Health Organisation. Let’s flatten the infodemic curve [Internet]. 2022 [accessed 04 August, 2022]. Available from: https://www.who.int/news-room/spotlight/let-s-flatten-the-infodemic-curve

Carlson CR, Rousselle H. Report and repeat: Investigating Facebook’s hate speech removal process. *First Monday*. 2020;25(2). DOI: 10.5210/FM.V25I2.10288

Merchant B. The promise of free speech on Elon Musk’s Twitter is officially dead [Internet]. [Online]. 2023. [Accessed 16 March, 2023]. Available from: https://www.latimes.com/business/technology/story/2023-03-06/column-the-promise-of-free-speech-on-elon-musks-twitter-is-officially-dead
